# Tracer Diffusion in Tightly-Meshed Homogeneous Polymer Networks: A Brownian Dynamics Simulation Study

**DOI:** 10.3390/polym12092067

**Published:** 2020-09-11

**Authors:** Hyun Woo Cho, Haein Kim, Bong June Sung, Jun Soo Kim

**Affiliations:** 1 Department of Chemistry, Sogang University, Seoul 04107, Korea; chohw2000@utexas.edu; 2 Department of Chemistry and Nanoscience, Ewha Womans University, Seoul 03760, Korea; khirfk@hanmail.net

**Keywords:** tracer diffusion, crosslinked network, Brownian dynamics, cage dynamics, hopping process

## Abstract

We report Brownian dynamics simulations of tracer diffusion in regularly crosslinked polymer networks in order to elucidate the transport of a tracer particle in polymer networks. The average mesh size of homogeneous polymer networks is varied by assuming different degrees of crosslinking or swelling, and the size of a tracer particle is comparable to the average mesh size. Simulation results show subdiffusion of a tracer particle at intermediate time scales and normal diffusion at long times. In particular, the duration of subdiffusion is significantly prolonged as the average mesh size decreases with increasing degree of crosslinking, for which long-time diffusion occurs via the hopping processes of a tracer particle after undergoing rattling motions within a cage of the network mesh for an extended period of time. On the other hand, the cage dynamics and hopping process are less pronounced as the mesh size decreases with increasing polymer volume fractions. The interpretation is provided in terms of fluctuations in network mesh size: at higher polymer volume fractions, the network fluctuations are large enough to allow for collective, structural changes of network meshes, so that a tracer particle can escape from the cage, whereas, at lower volume fractions, the fluctuations are so small that a tracer particle remains trapped within the cage for a significant period of time before making infrequent jumps out of the cage. This work suggests that fluctuation in mesh size, as well as average mesh size itself, plays an important role in determining the dynamics of molecules and nanoparticles that are embedded in tightly meshed polymer networks.

## 1. Introduction

The diffusion of a tracer particle in polymer networks has been under extensive investigation in various areas of science and engineering [1,2,3,4]. The measurement of tracer diffusion provides important pieces of microscopic information on the structural and dynamic properties of underlying polymeric materials, including polymer films [5,6,7,8,9,10], and biological cells [11,12,13,14,15], and on the penetration and mobility of analytes in polymer gels employed in a broad range of applications for drug delivery and sensors [9,16,17]. Among polymer networks, stimuli-responsive hydrogels serve as an interesting platform for the study of tracer diffusion [7,8,9,18,19], because the internal structure of the polymer networks can be altered significantly upon changes in environmental conditions, such as temperature, pH, ionic strength, and solvent quality [20,21,22]. However, the dynamics of molecules and nanoparticles in hydrogels is not well understood at the nanoscopic and mesoscopic scales, particularly with respect to the effect of change in the internal structure of the polymer network on molecular transport.

The porosity of polymer networks is a key parameter characterizing the internal structure of polymer networks and it is measured by a structural quantity, called the average mesh size, ξ [23]. Accordingly, previous studies on tracer diffusion in polymer networks have focused on understanding the effect of different network mesh sizes [7,8,9,19,24,25,26,27,28,29,30,31]. For instance, recent experiments on tracer diffusion in hydrogels investigated the effect of changes in ξ by varying the degree of crosslinking and by volume phase transition [7,8,9,19,26,31]. ξ decreases with an increasing degree of crosslinking and with a decrease in swelling ratio, and it was shown that the diffusion of a tracer particle in hydrogels, with variable crosslinking or swelling, is largely determined by ξ relative to the size of a tracer particle $\sigma_{tr}$, i.e., $\xi /\sigma_{tr}$. However, the systematic and quantitative investigation on the relation between tracer diffusion and the ratio of $\xi /\sigma_{tr}$ has been complicated due to the heterogeneity inherent in the polymer networks during the course of crosslinking or volume phase transition [19,31,32]. Computer simulations of polymer models, on the other hand, allow for the preparation of regularly crosslinked, homogeneous polymer networks and the investigation of tracer diffusion in the absence of network heterogeneity.

**Figure 1:**
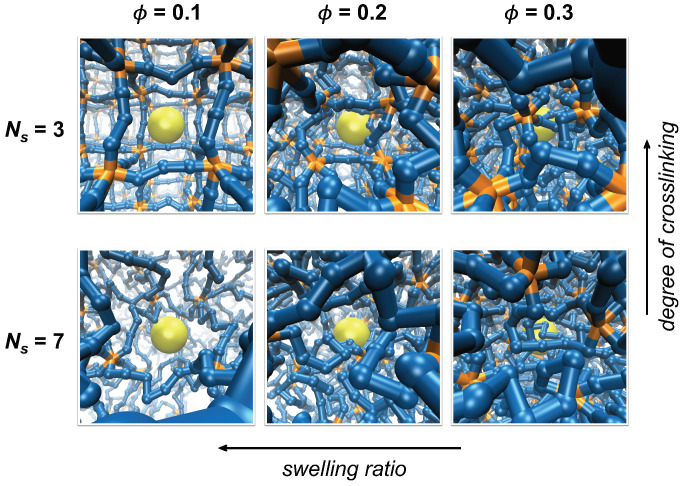
Simulation systems with varying degrees of crosslinking and swelling. The degree of crosslinking decreases as the number of polymer segments between crosslinks ($N_{s}$) increases while swelling ratio decreases as the volume fraction of polymer network (ϕ) increases. Segments composing the polymer network are colored blue, crosslinks are orange, and a tracer particle is yellow. Polymer segments are scaled down to half of the actual size for visualization.

In this work, we perform Brownian dynamics (BD) simulations of a tracer particle in a regularly crosslinked polymer network, i.e., in the model of a homogeneous polymer network to avoid the complexity that arises from network heterogeneity. In order to compare the tracer diffusion in polymer networks with various porosity, the network models with different mesh sizes are considered in this work by varying the degree of crosslinking and swelling in polymer networks, as shown in Figure 1 and Figure S1 of the Supplementary Material. The degree of crosslinking, referring to the fraction of crosslinking segments over all polymer segments, is adjusted by constructing the networks with different numbers of polymer segments between crosslinks and the swelling is by changing the network volume. Average mesh size, ξ, is calculated for each polymer network and the dynamics of a tracer particle is compared as a function of $\xi /\sigma_{tr}$. Although several factors influence the dynamics of a tracer particle in polymer networks, such as interactions between a tracer particle and polymer segments [8,19,33], aggregation of polymer segments of the network [30], and network heterogeneity [19,31,32], we solely focus on the size-dependent obstructive effect of small meshes by investigating the diffusion of an inert, nonsticky tracer particle in homogeneous polymer networks.

Similar with those in polymer solutions and melts [24,34,35], the dynamics of a tracer particle in polymer networks can be distinguished by the relative mesh size to that of a tracer particle, $\xi /\sigma_{tr}$ [36]. For large $\xi /\sigma_{tr}$, the network confinement has a negligible influence on tracer diffusion with the mean-square displacement (MSD) proportional to time (MSD∼$t^{1}$). When $\xi /\sigma_{tr}$ is close to the critical value, the dynamics of tracer particles is strongly coupled with the structure and dynamics of the network meshes [37,38]: the tracer particles exhibit subdiffusion at intermediate time scales (MSD∼$t^{\alpha }$ with α<1) and normal diffusion (MSD∼$t^{1}$) at longer times. However, unlike the polymer solutions and melts in which long-time diffusion occurs via the relaxation of surrounding polymer chains, long-time diffusion in polymer networks only occurs via the hopping process across the confining mesh [38,39]. For $\xi /\sigma_{tr}$ much smaller than a critical value, tracer particles are trapped and immobilized within a mesh, and the MSD does not increase with time (MSD∼$t^{\alpha }$ with α≪1).

Several computational studies have been reported to understand the dynamics of a tracer particle in polymer networks with permanent crosslinks [36,37,40,41,42,43,44,45]. In these works, diffusive behaviors of a tracer particle have been investigated at various values of $\xi /\sigma_{tr}$, either by changing the size of a tracer particle or changing the mesh size. They confirmed the above mentioned dependence of the tracer diffusion on the scale of $\xi /\sigma_{tr}$, and also showed that the dynamic fluctuations in polymer networks play an important role in the hopping motion of a tracer particle, especially when its size is comparable to or larger than the mesh size [37,40,42,44,45]. As for a computational model of the polymer network, lattice models have frequently been employed to mimic regular arrangement of crosslinks [36,37,40,43,44]. However, more realistic models have recently been employed by representing network strands with flexible polymer chains between crosslinks [42,45]. The network model employed in the present work is similar with that of Chen et al. [45] and polymer networks with different mesh sizes can be prepared by including different numbers of polymer segments between crosslinks, which mimics the variation in the degree of crosslinking. However, unlike the previous works, we also change polymer volume fractions as an additional means to change the network mesh size, which roughly mimics the volume change in swelling or collapse.

The average mesh size, ξ, is estimated in this study by the average distance between two connected crosslinks and ranges between 0.90$\sigma_{tr}$ and 1.84$\sigma_{tr}$, which are comparable to the size of a tracer particle. In all network conditions, the MSD results show subdiffusion of a tracer particle at intermediate time scales and normal diffusion at longer time scales. However, the duration of the subdiffusive regime is significantly prolonged as the average mesh size decreases with increasing degree of crosslinking, indicating more effective localization/trapping of a tracer particle within small meshes. Strong effects of localization/trapping by small network meshes are further examined by calculating the self-part of van Hove correlation functions and intermediate scattering functions as well as by directly observing particle displacements during simulation trajectories.

The average mesh size of the polymer network in this work is also decreased by increasing the polymer volume fraction at constant degree of crosslinking, causing interesting effects on tracer diffusion. Obviously, tracer diffusion becomes slower at higher polymer volume fractions, because of stronger obstruction by a high concentration of polymer segments near the tracer particle. However, the effect of the localization/trapping of a tracer particle that promotes subdiffusive behavior is less pronounced at higher polymer volume fractions, which is somewhat counterintuitive with respect to the stronger obstruction effect at higher volume fractions. We discuss the possible role of the restraints that are imposed by permanent crosslinks on conformational relaxation of network strands and verify that the restraints are stronger at lower polymer volume fractions by comparing fluctuations of network mesh size. We conclude that smaller fluctuation in mesh size at lower polymer volume fractions leads to more effective localization of a tracer particle within the cage of network meshes, for which long-time diffusion occurs via infrequent jumps out of the cage. The localized motion of a tracer particle within the cage and long-time diffusion via hopping at times are referred to as the cage dynamics and hopping process, respectively, hereafter in this work.

Finally, we present an example of two different network conditions, suggesting that the difference in transport mechanisms is not well described by the ratio of $\xi /\sigma_{tr}$. In this example, the average mesh sizes of the polymer networks and diffusion coefficients of the tracer particles are similar, but the transport mechanisms for tracer diffusion are quite distinct; one with normal Brownian diffusion and the other with hopping processes between neighboring meshes. Our results suggest that the fluctuation in mesh size plays an important role in determining how a tracer particle moves in tightly-meshed homogeneous polymer networks. In real polymer gels, the fluctuation in mesh size can be controlled by various factors including crosslinking density, swelling ratio of polymer gels, solvent conditions, and temperature. Therefore, our finding that the fluctuation in mesh size has a significant influence on tracer diffusion may then be used to finely control the transport of a tracer particle embedded in polymer gels. Additionally, synthetic methods for the formation of homogeneous polymer gels were recently reported [46]. Therefore, our work can also provide insights into tracer diffusion in several applications employing the homogeneous polymer gels.

The rest of this paper is organized, as follows. We describe the simulation model and method in Section 2, where we also discuss the average mesh size of various polymer networks employed in this work. Section 3 presents the simulation results, where we discuss reduced tracer diffusion with decreasing average mesh size, the hopping dynamics of a tracer particle in polymer networks with small mesh sizes, and then the effect of mesh size fluctuation on the transport mechanisms of a tracer particle. Section 4 summarizes this work.

## 2. Materials and Methods

Homogeneous polymer networks are modeled as regularly crosslinked and self-connected linear chains under periodic boundary conditions, as shown in Figure S1 of the Supplementary Material. Linear chains are modeled as a bead-spring chain, in which spherical segments are bonded together by a combination of the finite extension nonlinear elastic (FENE) potential

(1) $$U_{b}\left(r\right)=-\frac{1}{2}k_{b}R_{b}^{2}\;\ln\;\left[1-{\left(\frac{r}{R_{b}}\right)}^{2}\right]$$

and the repulsive part of the Lennard-Jones (LJ) potential

(2) $$U_{r}\left(r\right)=\left\{\begin{array}{ccc} 4\epsilon \left[{\left(\frac{\sigma }{r}\right)}^{12}-{\left(\frac{\sigma }{r}\right)}^{6}\right]+\epsilon & \; & 0<r<r_{c}\\ 0 & \; & \mathrm{elsewhere} \end{array}\right.$$

where $k_{b}=30k_{B}T/\sigma^{2}$, $R_{b}=1.5\sigma$, $\epsilon =1k_{B}T$, and the cut-off distance $r_{c}=2^{1/6}\sigma \approx 1.1225\sigma$. Here, $k_{B}$ is the Boltzmann constant and *T* is the temperature. The parameters $k_{b}$ and $R_{b}$ were chosen to prevent bond crossing [47], while $r_{c}$ is used to include only the repulsive part of the LJ potential. σ is approximately the diameter of each spherical polymer segment. The interaction between polymer segments is purely repulsive by employing the same repulsive LJ potential, as shown in Equation (2), to model the excluded volume interaction between polymer segments. Subsequently, the linear chains are crosslinked regularly at every $\left(N_{s}+1\right)$ segments along each chain, where $N_{s}$ is the number of polymer segments between two connected crosslinks. To form a large-scale polymer network, the periodic boundary condition is applied in all directions and both ends of each linear chain are also bonded together by the FENE and LJ potentials, resulting in all linear chains in the network being connected as a whole [48].

Polymer networks with different average mesh sizes are prepared by adjusting the number of permanent crosslinks and the swelling of the polymer networks. Networks are either constructed with different values of $N_{s}$ ranging between 3 and 9 for varying degrees of crosslinking, or they are compressed in order to achieve different degrees of network swelling and to set polymer volume fractions ϕ at different values of 0.1, 0.2, and 0.3, as shown in Figure 1 and Figure S1 of the Supplementary Material. The variation in crosslinking by changing $N_{s}$ mimics the formation of polymer networks at different concentrations of crosslinking agents or with different doses of crosslinking stimuli, such as UV irradiation. On the other hand, the variation in network swelling by changing ϕ is to roughly mimic the swelling behavior observed in hydrogels in response to external conditions, such as pH, temperature, and solvent quality [7,8,31]. A caveat of this approach is that the variation in swelling ratio in this work is induced by changing the system volume while keeping the number of polymer segments constant, whereas those induced experimentally occur by changing the effective interactions between polymer segments. The length of each linear chain is set as six to eight multiples of $\left(N_{s}+1\right)$ segments such that a unit simulation cell contains a large number of network meshes. Because the transport of a tracer particle is significantly influenced by the concentration of polymer segments, the effect of crosslinking is investigated at the same polymer volume fraction, ϕ, for various values of $N_{s}$ by compressing the system volume. Therefore, the network mesh sizes are adjusted either by changing $N_{s}$ at the same ϕ or by changing ϕ at the same $N_{s}$.

The network model employed by Chen et al. [45] seems very appealing because the formation of the polymer network through the association of precursor chains with multifunctional junctions mimics experimental procedures. However, the tracer diffusion was only examined after the network formation was completed and there are several approximations in their model that may affect the network structure, including the use of a simple reaction scheme for crosslinking. The perfect network model employed in this work provides an ideal platform for the study of the tracer dynamics without any model-dependent structural variation. In addition, the focuses of the works are also slightly different. They used a wide range of $N_{s}$ (=5∼200) at constant ϕ to examine the tracer diffusion both in unentangled and entangled networks. On the other hand, we only focus on the unentangled polymer network with the average mesh size being comparable to the size of a tracer particle. The mesh sizes are controlled either by changing $N_{s}$ (=3∼9) at constant ϕ or by changing ϕ at constant $N_{s}$.

Tracer diffusion in polymer networks is investigated for a spherical tracer particle with a size $\sigma_{tr}$, which is comparable to the average mesh sizes of the polymer networks. Because the smallest mesh size of the polymer networks is attained for $N_{s}=3$, the diameter of the tracer particle is set to $\sigma_{tr}=3\sigma$, such that the fully stretched polymer network has a pore size similar to the diameter of the tracer particle. The interaction between a tracer particle and the polymer segments is also purely repulsive by employing the modified LJ potential, as below.

(3) $$U_{r}\left(r\right)=\left\{\begin{array}{ccc} 4\epsilon \left[{\left(\frac{\sigma }{r-r_{s}}\right)}^{12}-{\left(\frac{\sigma }{r-r_{s}}\right)}^{6}\right]+\epsilon & \; & r_{s}<r<r_{s}+r_{c}\\ 0 & \; & \mathrm{elsewhere} \end{array}\right.,$$

where $r_{s}$ is the value shifting the LJ potential and is set 1σ, so that a tracer particle with a diameter of 3σ and a polymer segment with a diameter of 1σ repel each other when their distance is less than $r_{s}+r_{c}=\left(1+2^{1/6}\right)\sigma \approx 2.1225\sigma$.

The dynamic motions of a tracer particle and the polymer segments are described by the position Langevin equation, as shown in Equation (4), in order to mimic the solvent-induced Brownian motions of particles without direct incorporation of solvent molecules. The BD simulations are performed by numerically solving Equation (4) using GROMACS v. 4.5.4 [49]. At each time step, Δt, the position ${\vec{r}}_{i}\left(t\right)$ of a particle *i* (either a tracer particle or polymer segments) is updated via

(4) $${\vec{r}}_{i}\left(t+\Delta t\right)={\vec{r}}_{i}\left(t\right)+\frac{D_{i}^{0}{\vec{F}}_{i}\left(t\right)}{k_{B}T}\Delta t+{\vec{R}}_{i}\left(\Delta t\right)$$

where ${\vec{F}}_{i}\left(t\right)$ is the total force acting on particle *i* and ${\vec{R}}_{i}\left(\Delta t\right)$ is a random displacement with a Gaussian distribution function exhibiting zero mean and variance-covariance of $\langle {\vec{R}}_{i}\left(\Delta t\right){\vec{R}}_{j}\left(\Delta t\right)\rangle =6D_{i}^{0}\Delta t{\vec{\delta }}_{ij}$. The diffusion coefficient, $D_{i}^{0}$, of each polymer segment is set equal to a value of $D_{0}$, which sets the time scale by defining the time unit of $\tau_{BD}=\sigma^{2}/D_{0}$. For a tracer particle, $D_{i}^{0}=D_{0}/3$ based on the size difference. A time step of $\Delta t=10^{-4}\;\tau_{BD}$ is used for all simulations. A total of $10^{5}\tau_{BD}$ is performed for each simulation with data recording at every 10$\tau_{BD}$. Data at shorter time scales are obtained by performing additional short simulations with a duration of $2\times 10^{2}\tau_{BD}$ and with data recording at every $10^{-2}\tau_{BD}$. The total force ${\vec{F}}_{i}\left(t\right)$ is calculated from the bonded and non-bonded interactions described in Equations (1)–(3). For each polymer network with $\left(\phi ,N_{s}\right)$, five independent simulations are performed to calculate the statistical properties. Hydrodynamic interactions are not considered in this work.

**Figure 2:**
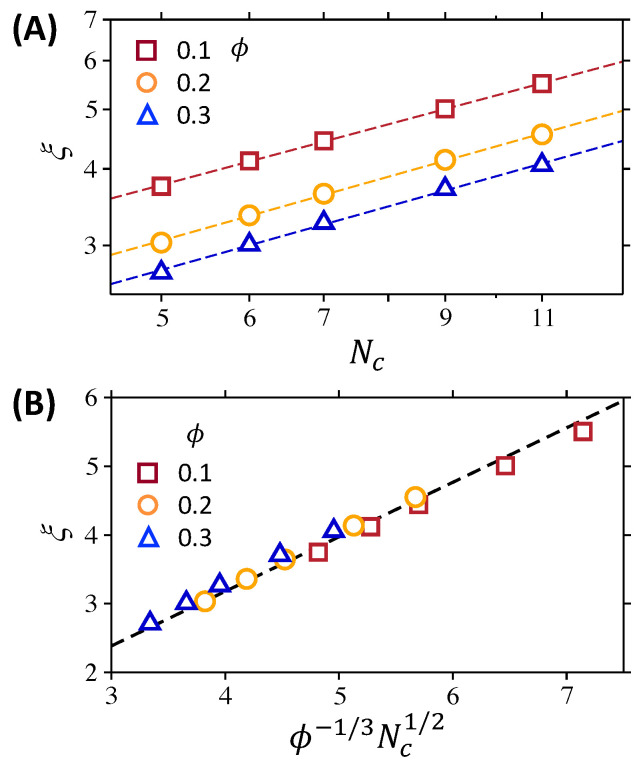
(**A**) Average mesh size ξ of polymer networks as a function of $N_{c}$ for various ϕ, where $N_{c}$ is the number of polymer segments including those at both crosslinking sites, such that $N_{c}=N_{s}+2$. The symbols are the values of ξ calculated from simulations and the corresponding dashed lines are drawn to guide the eyes. (**B**) ξ at various $N_{c}$ and ϕ as a function of $\phi^{-1/3}N_{c}^{1/2}$. The black dashed line confirms the scaling relation of ξ, i.e., ξ∼$\phi^{-1/3}N_{c}^{1/2}$.

The average mesh size is used to characterize the pore structure of various polymer networks. Several definitions of average mesh size, ξ, are used depending on experimental approaches [23]. We calculate the distance between two connected crosslinks to define the geometric mesh size. The estimated average mesh size ranges between 2.71σ and 5.51σ; thus, $\xi /\sigma_{tr}$ ranges between 0.90 and 1.84, so that the size of a tracer particle is comparable to average mesh size. In Figure 2A, the average mesh size is presented as a function of $N_{c}$, where $N_{c}$ is the number of polymer segments including those at both crosslinking sites, such that $N_{c}=N_{s}+2$. The average mesh size increases with $N_{c}$ at the same ϕ, but decreases with ϕ at the same $N_{c}$. The scaling relation of average mesh size with ϕ and $N_{c}$ can be expressed as

(5) $$\xi ∼\phi^{-1/3}{\left(r_{0}^{2}\right)}^{1/2}∼\phi^{-1/3}N_{c}^{1/2},$$

where $r_{0}$ is the distance between two connected crosslinks. This equation is derived by assuming the affine polymer network with ideal network segments [50]. Figure 2B shows that the average mesh sizes of all polymer networks presented as a function of $\phi^{-1/3}N_{c}^{1/2}$ collapse into a single line, consistent with Equation (5).

## 3. Results and Discussion

### 3.1. Effect of Variation in the Degree of Crosslinking

**Figure 3:**
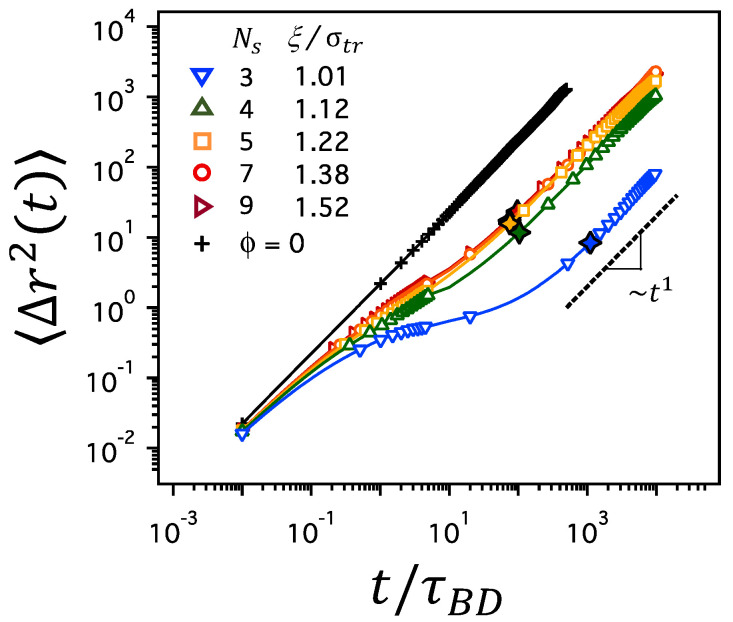
MSD, $\langle \Delta r^{2}\left(t\right)\rangle$, for various values of $N_{s}$ and corresponding $\xi /\sigma_{tr}$ at ϕ=0.2, where $\sigma_{tr}$ is the diameter of a tracer particle. The line of ∼$t^{1}$ (the black dashed line) is the guide to the eyes denoting the diffusive, linear regime at long times ($t>\tau_{D}$), where $\tau_{D}$ is the crossover time distinguishing subdiffusion and normal diffusion and marked by black open diamonds. The MSD of a tracer particle in free solutions without polymer network is also presented for comparison (black plus symbols marked as ϕ=0).

As the average mesh size, ξ, of the polymer network decreases, either by a decrease in $N_{s}$ at constant ϕ or by an increase in ϕ at constant $N_{s}$, the dynamics of a tracer particle in the polymer network slows down. Figure 3 depicts the MSD, $\langle {\left(\vec{r}\left(t\right)-\vec{r}\left(0\right)\right)}^{2}\rangle \equiv \langle \Delta r^{2}\left(t\right)\rangle$, for various values of $N_{s}$ and corresponding mesh sizes of $\xi /\sigma_{tr}$ at a constant polymer volume fraction of ϕ=0.2. In a free solution without a polymer network (plus symbols marked with ϕ=0), the MSD is linear throughout the time regime between $10^{-2}\tau_{BD}$ and $10^{3}\tau_{BD}$. However, in polymer networks, the MSD increases linearly at short times, grows more slowly at intermediate time scales, and reaches the linear regime at longer times, which results in the overall delay of tracer diffusion in polymer networks. The delay is more significant when $N_{s}$ is as small as 3, which corresponds to a mesh size as small as $\xi /\sigma_{tr}=1.01$. The slow growth of MSD at intermediate time scales suggests the subdiffusion that follows the relation of $\langle \Delta r^{2}\left(t\right)\rangle$∼$t^{\alpha }$ with α<1, whereas the linear growth of MSD at longer times suggests normal Brownian diffusion with $\langle \Delta r^{2}\left(t\right)\rangle$∼$t^{1}$. Here, we define the characteristic time scale, $\tau_{D}$, of a tracer particle moving with the diffusion coefficient *D* by a distance of average mesh size, ξ, such that $\tau_{D}=\xi^{2}/6D$. The diffusion coefficient, *D*, is estimated by the long-time behavior of MSD as $\lim_{t\to \infty }\langle \Delta r^{2}\left(t\right)\rangle /6t$. In Figure 3, $\tau_{D}$ is marked with black open diamonds. $\tau_{D}$ clearly separates the time regimes with different scalings of MSD and it is used as the crossover time distinguishing between subdiffusion and normal Brownian diffusion.

It is noted in Figure 3 that the duration of the subdiffusive regime is prolonged as the average mesh size, ξ, decreases at constant ϕ. At ϕ=0.2, the time regime for subdiffusion is the longest for the network with $N_{s}=3$ and shorter for the networks with larger $N_{s}$ values. In particular, the MSD nearly flattens at intermediate time scales in the network with $N_{s}=3$. These results are consistent with those reported in previous works [45]. The same observation holds at different ϕ’s, as shown for ϕ=0.1 and 0.3 in Figure S2 of the Supplementary Material. At small values of $N_{s}$ corresponding to high degree of crosslinking (for which the contour length of each network strand becomes comparable to or as short as the size of a tracer particle), the tracer particle is caged tightly within the pore space of the confining mesh. Thus, the movement from one pore space to another is severely restrained, delaying the growth of $\langle \Delta r^{2}\left(t\right)\rangle$ for a significant period of time before reaching the time regime for normal diffusion.

Analysis of MSD alone, albeit informative, does not distinguish different mechanisms of restricted diffusion [51]. In order to account for the effect of crosslinking on the dynamics of a tracer particle in polymer networks, we examine the motion of a tracer particle more closely. We first calculate the self-part of the van Hove correlation function, $G_{s}\left(r,t\right)$, defined as $\langle \delta \left\{r-\left[\vec{r}\left(t\right)-\vec{r}\left(0\right)\right]\right\}\rangle$, where $\left[\vec{r}\left(t\right)-\vec{r}\left(0\right)\right]$ is the positional displacement of a tracer particle over a time interval of *t* [52,53]. $G_{s}\left(r,t\right)$ estimates the distribution of particle displacements during time *t*. For a particle undergoing random Brownian displacements, $G_{s}\left(r,t\right)$ can be expressed as the Gaussian distribution,

(6) $$G_{s}\left(r,t\right)={\left(\frac{1}{4\pi Dt}\right)}^{3/2}\exp\left[-\frac{r^{2}}{4Dt}\right].$$

**Figure 4:**
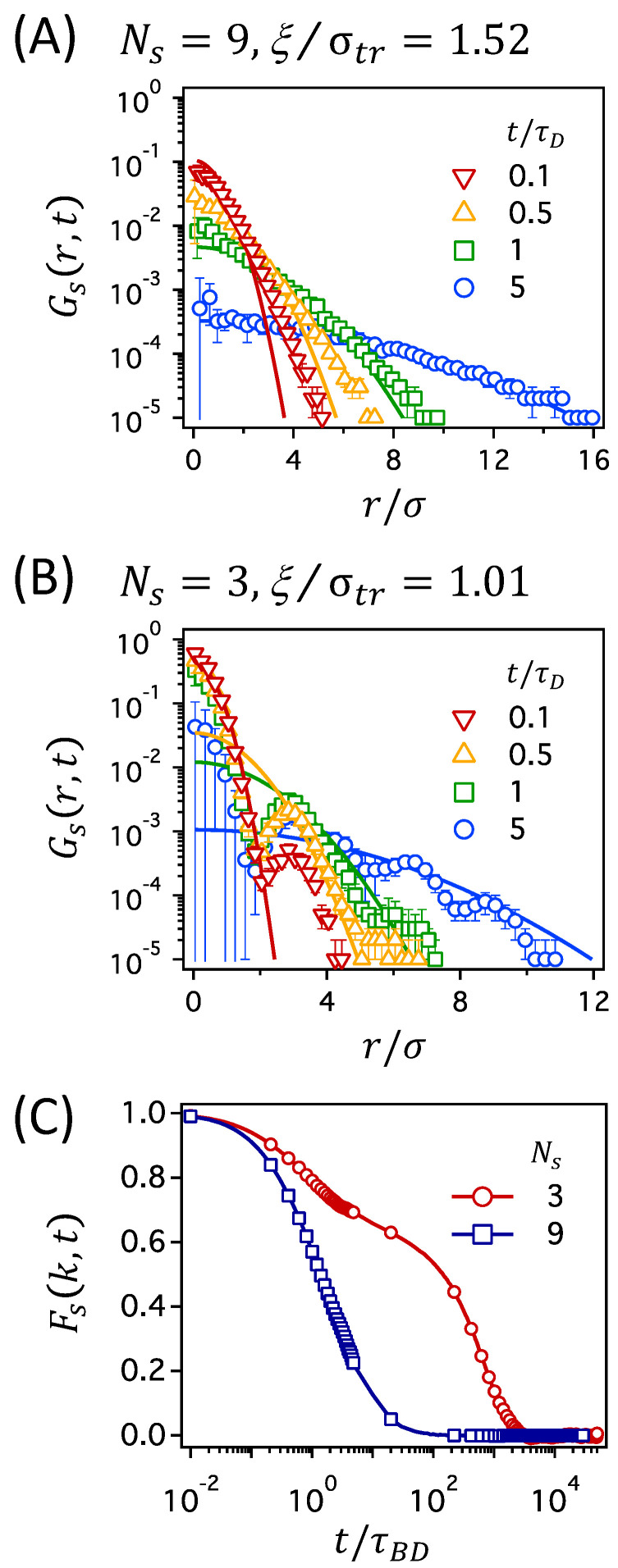
$G_{s}\left(r,t\right)$ as a function of r/σ at different times $t/\tau_{D}$ for (**A**) $N_{s}=9$, or equivalently $\xi /\sigma_{tr}=1.52$ and (**B**) $N_{s}=3$, or equivalently $\xi /\sigma_{tr}=1.01$ at the polymer volume fraction of ϕ=0.2. The self-part of intermediate scattering function $F_{s}\left(k,t\right)$ is presented in (**C**) for the two network conditions. Solid curves in (**A**,**B**) represent the Gaussian distribution of Equation (6) calculated with the diffusion coefficient *D* calculated from a long-time behavior of MSD in Figure 3. In (**C**), $F_{s}\left(k,t\right)$ is calculated using the value of k=2π/3 that corresponds to the diameter of a tracer particle.

In Figure 4, we present $G_{s}\left(r,t\right)$ for a tracer particle in two polymer networks at constant ϕ=0.2: one with the largest mesh size of $N_{s}=9$ (corresponding to the lowest degree of crosslinking in this work) in panel (A) and the other with the smallest mesh size of $N_{s}=3$ (corresponding to the highest degree of crosslinking) in panel (B). In the figure, several data of $G_{s}\left(r,t\right)$ are presented at different times ranging from $t=0.1\tau_{D}$ to $5\tau_{D}$, which cover the time regimes for both subdiffusion and normal diffusion. Solid lines in Figure 4A,B are the Gaussian distributions that are calculated using Equation (6), for which the diffusion coefficient *D* is calculated from the long-time behavior of MSD presented in Figure 3. In Figure S3 of the Supplementary Material, the figures of $4\pi r^{2}G_{s}\left(r,t\right)$ corresponding to $G_{s}\left(r,t\right)$ in Figure 4A,B are presented.

The results presented in Figure 4A,B suggest that the dynamics of a tracer particle can be qualitatively different for polymer networks with different degrees of crosslinking. It is shown that the data of $G_{s}\left(r,t\right)$ for a tracer particle in the network with $N_{s}=9$ agree well with the Gaussian distributions, whereas those in the network with $N_{s}=3$ deviate significantly from the Gaussian distributions. It is noted that the Gaussian distributions, as shown as solid curves in Figure 4, are directly calculated from Equation (6) using the long-time data of MSD (from which the diffusion coefficient is calculated) and are not determined by fitting Equation (6) to the simulation data of $G_{s}\left(r,t\right)$. Given that, the agreement of $G_{s}\left(r,t\right)$ with the Gaussian distributions for the network with $N_{s}=9$ in Figure 4A is fairly good and suggests that a tracer particle behaves according to normal Brownian diffusion at all times.

However, in the network with $N_{s}=3$, $G_{s}\left(r,t\right)$ is non-Gaussian showing significant deviations from the Gaussian distributions, as shown in Figure 4B. The most prominent difference is the oscillatory decay of $G_{s}\left(r,t\right)$ with distance *r*, compared to the monotonous decay of the Gaussian distribution: small peaks develop at several values of *r* in addition to the major peak around r=0, which becomes more evident at longer times. The second prominent feature is that the height of the first peak does not diminish significantly with time. These non-Gaussian features for the polymer network with the smallest mesh size suggest that the tracer particle is caged and severely restrained within the pore space of the confining mesh (based on the perseverance of the first peak). Moreover, the long-time particle displacements occur by hopping processes (evidenced by the appearance of multiple peaks at large *r*), which is consistent with earlier understandings that the hopping is the only possible mechanism for tracer diffusion in crosslinked polymer networks with small mesh sizes [38,39]. Here, we emphasize that the deviation of $G_{s}\left(r,t\right)$ from the Gaussian distribution is not reduced at long times, $t>\tau_{D}$, where MSD becomes linear with time, suggesting the normal Brownian dynamics (following the Fick’s law of diffusion). This serves as an example of Fickian yet non-Gaussian dynamics [53,54,55].

Such Fickian yet non-Gaussian dynamics, which has been broadly observed in dense colloidal suspensions, cell cytoplasm, membrane, and entangled actin filaments [14,53,54,55,56,57], was suggested as a signature of the hopping motions of a tracer particle in confined diffusion [58]. In this work, the hopping processes of a tracer particle, suggested above based on the oscillatory shape of $G_{s}\left(r,t\right)$, can be directly confirmed by observing particle displacements in simulated trajectories, as shown in Figure S4 of the Supplementary Material. For the network with large meshes at $N_{s}=9$, the position of a tracer particle changes constantly and continuously, confirming Brownian dynamics. On the other hand, the position of a tracer particle in the network with small meshes at $N_{s}=3$ changes intermittently and discretely, indicating the hopping processes of a tracer particle between neighboring meshes after a long period of rattling motions in a single cage.

The dynamic behaviors of a tracer particle can also be distinguished by the self-part of the intermediate scattering function $F_{s}\left(k,t\right)$, as shown in Figure 4C. $F_{s}\left(k,t\right)$ describes the density correlation of a tracer particle over the time interval of *t* on the length scale of $k^{-1}$ and, thus, the decay of $F_{s}\left(k,t\right)$ with time is related to the particle motions on the length scale [53,59,60,61]. $F_{s}\left(k,t\right)$ can be calculated from the positions of a tracer particle in simulation trajectories as $F_{s}\left(k,t\right)=\langle \exp\left[i\vec{k}\cdot \left(\vec{r}\left(t\right)-\vec{r}\left(0\right)\right)\right]\rangle$, where *k* is the modulus of the wave vector $\vec{k}$. In this work, we use the value of k=2π/3, which corresponds to the diameter of a tracer particle. In the network with large meshes at $N_{s}=9$, $F_{s}\left(k,t\right)$ decays smoothly down to zero with time. In the network with small meshes at $N_{s}=3$; however, the initial decay of $F_{s}\left(k,t\right)$ slows down at intermediate times, followed by the second relaxation to zero. While the single decay of $F_{s}\left(k,t\right)$ in the network with $N_{s}=9$ suggests normal Brownian diffusion of a tracer particle, the two-step decay in the network with $N_{s}=3$ is characteristic of cage dynamics and hopping processes of a tracer particle and, thus, describe the motions of the tracer particle rattling around within the cage at intermediate times and making infrequent jumps out of the cage at long times.

### 3.2. Effect of Variation in Polymer Volume Fraction

**Figure 5:**
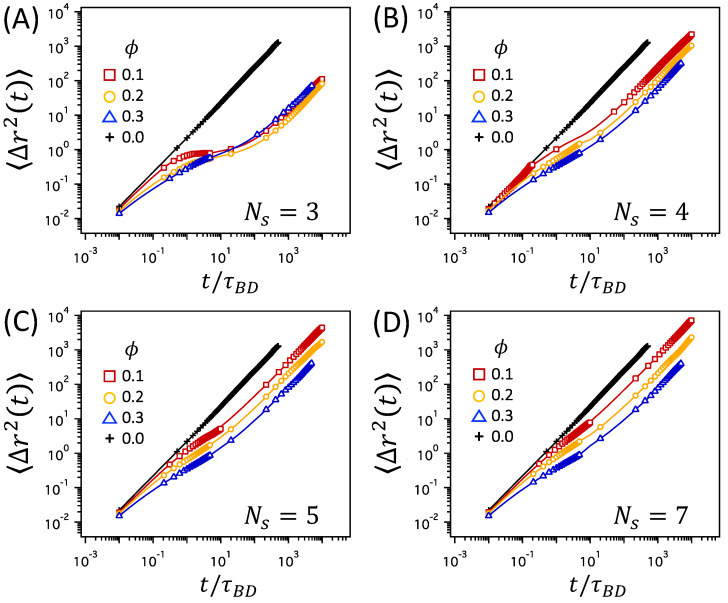
MSD, $\langle \Delta r^{2}\left(t\right)\rangle$, for different values of ϕ at $N_{s}=3$, 4, 5, and 7 in panels (**A**–**D**).

Thus far, we have discussed the effect of crosslinking by studying several polymer networks with various $N_{s}$ values at constant ϕ=0.2. Here, we examine the effect of varying the polymer volume fraction, ϕ, at the same degree of crosslinking of constant $N_{s}$. This roughly mimics the structural changes in polymer networks induced by the volume change of swelling or collapse. Figure 5 compares the MSD data for different ϕ (also shown in Figure 3 and Figure S2 of the Supplementary Material) and reveals two notable effects of changing ϕ. First, as ϕ increases for polymer networks with the same $N_{s}$, the MSD data shift away from that of the free solution (marked with ϕ=0) towards longer times, which implies that, on average, it takes longer at higher ϕ for a tracer particle to move by the same distance (except for the network with $N_{s}=3$ at ϕ=0.3). The change is particularly clear at short times: at ϕ=0.1, the MSD data for the networks are almost indistinguishable from that of the free solution, whereas when ϕ increases, the MSD data deviate significantly from that of the free solution. Secondly, as ϕ increases, the subdiffusive regime becomes less clearly distinguished. For the networks with $N_{s}=3$, for instance, the MSD at ϕ=0.1 shows abrupt changes in the slope and forms a plateau at intermediate times before reaching the linear diffusive regime, whereas the MSD at ϕ=0.3 increases continuously with gradual changes in the slope from short to long times. The sharp changes in the slope for ϕ=0.1 suggest cage dynamics at intermediate times and hopping process at long times, as discussed above for the networks with high degree of crosslinking. On the other hand, although it is still densely crosslinked, the cage dynamics and hopping process are not clearly implicated at ϕ=0.3.

The former effect can be understood in terms of stronger obstruction by the increase in polymer volume fraction. As ϕ increases at constant $N_{s}$, the concentration of polymer segments increases near the tracer particle. Because the motions of the tracer particle are more severely obstructed in a more crowded neighborhood, the overall transport of the particle is slowed down and the MSD data shift towards longer times. However, the latter effect of changing ϕ is not fully understood in terms of stronger obstruction. In fact, it seems somewhat counterintuitive that the cage dynamics and hopping process are more clearly seen under less crowded network condition of ϕ=0.1 than under more crowded condition of ϕ=0.3. The cage dynamics and hopping process are, naively, more anticipated in more crowded environments, because the tracer particle is being caged for a long time before the surrounding polymer segments relax to make an open room for the tracer particle to escape.

**Figure 6:**
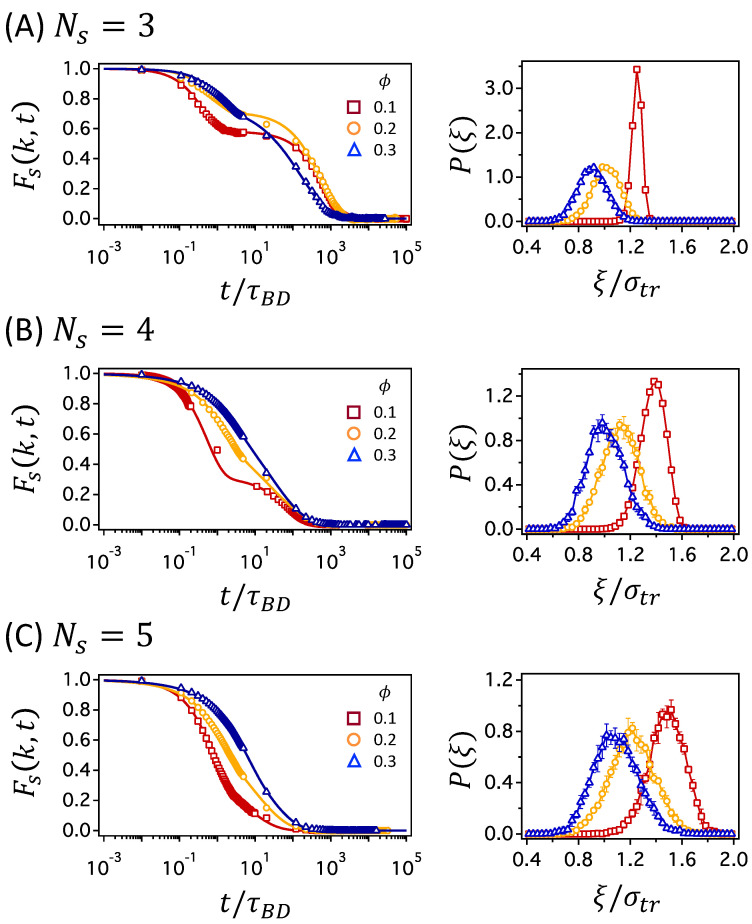
Self-part of intermediate scattering functions $F_{s}\left(k,t\right)$ (left panels) and probability distribution functions of mesh size P(ξ) (right panels), presented at various polymer volume fractions ϕ in networks with (**A**) $N_{s}=3$, (**B**) $N_{s}=4$, and (**C**) $N_{s}=5$.

In Figure 6, the time relaxations of $F_{s}\left(k,t\right)$ are compared at different ϕ values for networks with $N_{s}=3$, 4, and 5, in panels (A), (B), and (C), respectively, for which the number of crosslinks is the highest. The decay of $F_{s}\left(k,t\right)$ distinguishes the mechanisms of tracer diffusion, as mentioned earlier, depending on whether the function decays in a single step or two steps: the two-step decay is characteristic of cage dynamics at intermediate times and hopping process at long times, whereas the single decay results from normal Brownian diffusion. In Figure 6A for the network with $N_{s}=3$, the decay of $F_{s}\left(k,t\right)$ changes from the two-step process to the single-step process as ϕ increases. The cage dynamics and hopping process are more clearly seen under a less crowded network condition of ϕ=0.1 than under more crowded condition of ϕ=0.3. The change is moderate for networks with $N_{s}=4$, as shown in Figure 6B, where the two-step decay is clearly observed only at ϕ=0.1. For networks with $N_{s}=5$, the decays of all $F_{s}\left(k,t\right)$ values occur in a single step, which suggests that the cage dynamics and hopping process are less likely. We already drew the same conclusions as above by comparing the MSD data in Figure 3 and Figure S2 of the Supplementary Material. However, the comparison is clearer while using $F_{s}\left(k,t\right)$ for the networks with $N_{s}=4$ and 5 as the subdiffusive regime is not well distinguished in the MSD data.

For a tracer particle to move by a distance comparable to its size, or farther, the polymer segments surrounding the tracer particle have to relax to make room for the tracer particle to escape from the cage. As ϕ increases, the relaxation time of the polymer segments increases, and cage dynamics is more anticipated. However, the presence of permanent crosslinks in the polymer network subverts this belief. At low ϕ, network strands between crosslinks are stretched and the structural relaxation is hampered. Rather, at higher ϕ, network strands are more relaxed in the sense that the distance between neighboring crosslinks is shorter than its contour length and, thus, the cooperative structural change is viable to help a tracer particle escape from the cage. This conjecture is confirmed by examining the network fluctuations at various ϕ values, as shown in the right-hand panels of Figure 6A–C, which present the probability distribution of mesh size, ξ. As ϕ increases for networks with the same $N_{s}$, the distribution shifts to the left, and the average mesh size, ξ, decreases. However, the width of the distribution becomes wider as ϕ increases, which implies that the fluctuation in mesh size becomes larger at higher ϕ. This confirms the above idea that the network strands are more relaxed at higher ϕ, which leads to cooperative changes in the network structure, increasing the chance of the particle escaping from the cage. By calculating mesh size fluctuation for the networks where $F_{s}\left(k,t\right)$ decays in two steps, a rough estimate can be made for the critical size of the fluctuation that separates normal and hopping dynamics. The two-step decay of $F_{s}\left(k,t\right)$ is only observed when $\Delta \xi /\sigma_{tr}\leq 0.11$, where Δξ is the standard deviation of the ξ distribution. If the fluctuation is less than 11% of the particle diameter, a tracer particle is likely to be caged in the mesh for a significant period of time, which results in the rattling motion within the mesh and the jumps by a large distance out of the cage at times. If the fluctuation is larger, the dynamic motions of a tracer particle occur concurrently with the network fluctuation, such that the transport of a tracer particle occurs via normal Brownian diffusion.

### 3.3. Effect of Fluctuation in Mesh Size

**Figure 7:**
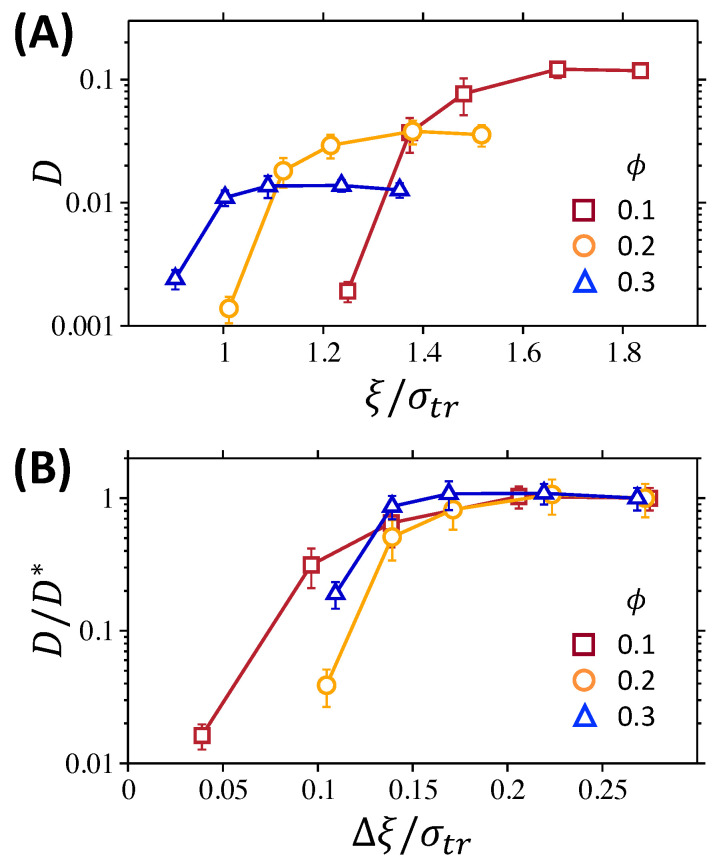
(**A**) Diffusion coefficient *D*, in unit of $\sigma^{2}/\tau_{BD}$, as a function of the relative mesh size $\xi /\sigma_{NP}$ at the polymer volume fractions ϕ=0.1, 0.2, and 0.3. (**B**) The ratio of diffusion coefficient *D* to that with the largest mesh size $D^{*}$ at the same volume fraction ϕ, as a function of the mesh size fluctuation $\Delta \xi /\sigma_{NP}$.

The overall effect of network confinement on tracer diffusion is summarized in Figure 7 in terms of the diffusion coefficient, *D*, for all network conditions. The diffusion coefficients are calculated from the MSDs in Figure 3 by $\lim_{t\to \infty }\langle \Delta r^{2}\left(t\right)\rangle /6t$. The relative size of the average mesh to the size of the tracer particles, $\xi /\sigma_{tr}$, has often been considered to be a governing factor that determines the transport of tracer particles in polymer networks. In Figure 7A, the diffusion coefficient is presented as a function of $\xi /\sigma_{tr}$ at different ϕ values. At each ϕ, the diffusion coefficient decreases as $\xi /\sigma_{tr}$ decreases. The decrease in *D* is minute at large $\xi /\sigma_{tr}$, corresponding to the network with $N_{s}=5$, 7, and 9, whereas the reduction is significant at smaller $\xi /\sigma_{tr}$, which corresponds to $N_{s}=3$ and 4. In particular, for the network with the smallest $\xi /\sigma_{tr}$ at each ϕ, the diffusion coefficient is less by an order of magnitude. However, no universal relation can be found between reduced tracer diffusion and relative mesh size, $\xi /\sigma_{tr}$. The dramatic reduction in tracer diffusion occurs at different $\xi /\sigma_{tr}$ values depending on the polymer volume fraction at $\xi /\sigma_{tr}$ between 1.25 and 1.37 for ϕ=0.10, between 1.01 and 1.12 for ϕ=0.20, and between 0.90 and 1.00 for ϕ=0.30. This observation is qualitatively consistent with the theoretical prediction by Dell and Schweizer [38] that the localization/trapping of a tracer particle and corresponding change in tracer diffusion occur at different critical values of $\xi /\sigma_{tr}$, depending on the specific properties of polymer networks, such as the network compressibility.

Alternatively, we present the diffusion coefficient as a function of the network fluctuation, $\Delta \xi /\sigma_{tr}$, in Figure 7B. Here, the diffusion coefficient, *D*, is normalized by the value $D^{*}$ of the network with the largest mesh size at each ϕ, such that $D/D^{*}$, at the largest mesh size, equals 1. Interestingly, the dependence of the diffusion coefficient $D/D^{*}$ on $\Delta \xi /\sigma_{tr}$ at various ϕ values is qualitatively in mutual agreement. The value remains similar at large $\Delta \xi /\sigma_{tr}$ values, whereas $D/D^{*}$ drops rapidly as $\Delta \xi /\sigma_{tr}$ decreases below 0.11. The condition of $\Delta \xi /\sigma_{tr}\leq 0.11$ for the dramatic reduction in diffusion is consistent with the network fluctuations that enable the two-step decay of $F_{s}\left(k,t\right)$, suggesting that the reduced diffusion is attributed to the transition from normal diffusive dynamics to the cage dynamics and hopping process of a tracer particle. Therefore, we conclude that the long-time dynamics of a tracer particle, as represented by the diffusion coefficient, is greatly influenced by the cage effect within the network mesh, which, in turn, is greatly influenced by the fluctuation in network meshes. The importance of fluctuations in network meshes has been discussed in previous works [37,39,40,42,44,45]. In previous studies using the lattice models, the elastic constant between crosslinks was adjusted to control the fluctuations in mesh sizes, and it was shown that, when the size of a tracer particle is comparable to the mesh size, MSD grows more slowly for the networks with smaller fluctuations [37,39,40,42,44]. In the work by Chen et al., significant increase in the crossover time from subdiffusive to normal diffusive behaviors for large tracers was associated with the decrease in mesh size fluctuations [45], supporting the argument that the fluctuation in network meshes plays an important role for the long-time dynamics of a tracer particle whose size is comparable to the mesh size.

**Figure 8:**
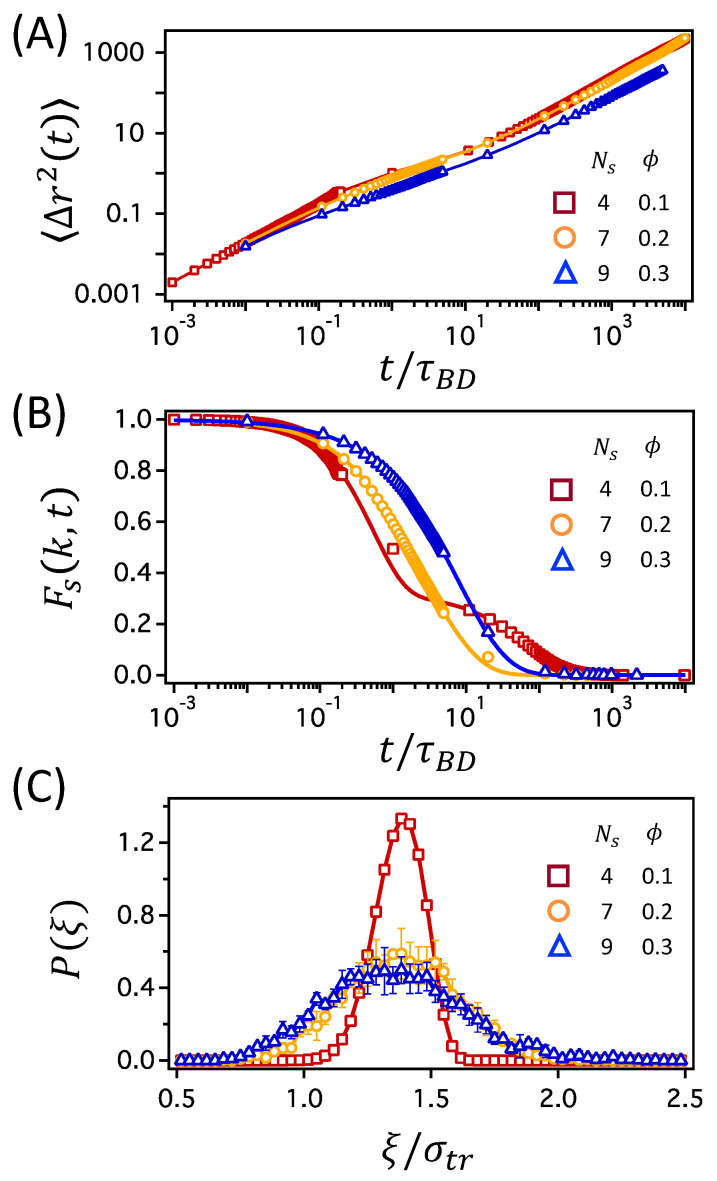
(**A**) MSD $\langle \Delta r^{2}\left(t\right)\rangle$, (**B**) self-part of the intermediate scattering function $F_{s}\left(k,t\right)$, and (**C**) the probability distribution P(ξ) for the three network conditions with similar values of the relative mesh size $\xi /\sigma_{tr}$: 1.37, 1.38, and 1.35 for the networks with $\left(N_{s},\phi \right)=\left(4,0.1\right)$, (7,0.2), and (9,0.3), respectively.

Finally, the importance of the fluctuations in mesh size is highlighted by comparing the three network conditions with similar values of $\xi /\sigma_{tr}≃1.37$. The mesh size corresponds to the networks with $N_{s}=4$ at ϕ=0.1, $N_{s}=7$ at ϕ=0.2, and $N_{s}=9$ at ϕ=0.3, as presented in Figure 7A. In the figure, the diffusion coefficients at ϕ=0.1 and 0.2 are almost the same, whereas that at ϕ=0.3 is smaller. Figure 8 presents MSD, $F_{s}\left(k,t\right)$, and P(ξ) for the three network conditions. In Figure 8A, the MSDs are very similar for the three network conditions. Because the diffusion coefficient at ϕ=0.3 is smaller than those for the other two conditions, the MSD at ϕ=0.3 also deviates slightly from the other two. However, no qualitative difference is observed in the MSDs. On the other hand, the intermediate scattering functions, $F_{s}\left(k,t\right)$, in Figure 8B are distinguishable from one another. In particular, $F_{s}\left(k,t\right)$ at ϕ=0.1 has a two-step decay, whereas those at ϕ=0.2 and 0.3 decay in a single step. This suggests cage dynamics and hopping process for a tracer particle at ϕ=0.1 and normal diffusive dynamics at higher ϕ values. Therefore, it can be concluded that the transport mechanisms are quite different for polymer networks at ϕ=0.1 and 0.2, even when their MSDs, diffusion coefficients, and $\xi /\sigma_{tr}$ are very similar. The difference in transport mechanisms can also be seen in the individual trajectories of a tracer particle shown in Figure S5 of the Supplementary Material: rattling and hopping at ϕ=0.1 and normal diffusion at ϕ=0.2 and 0.3.

The transport mechanisms of a tracer particle are better correlated with the mesh size fluctuation of $\Delta \xi /\sigma_{tr}$ rather than with the average mesh size, $\xi /\sigma_{tr}$. For the polymer networks at ϕ=0.2 and 0.3, in which the normal diffusion of a tracer particle is observed, P(ξ) in Figure 8C is broader with $\Delta \xi /\sigma_{tr}=0.22$ and 0.27, respectively. On the other hand, for the network at ϕ=0.1, in which the hopping dynamics is observed, P(ξ) has a narrower distribution with $\Delta \xi /\sigma_{tr}=0.10$. Movements of a tracer particle in networks with small mesh sizes occur collectively with the conformational fluctuations of the polymer segments in the meshes [37]. The small fluctuations in mesh size at ϕ=0.1 thus inhibit the diffusive movement of a tracer particle through meshes, resulting in the trapping at short times and hopping at longer times.

## 4. Conclusions

The transport of a tracer particle was investigated in tightly-meshed, homogeneous polymer networks with various mesh sizes that were comparable to the size of a tracer particle. As the average mesh size of the polymer network decreases, either by an increase in the degree of crosslinking or by an increase in polymer volume fraction, the diffusion of a tracer particle in the polymer networks slows down. In particular, at high degree of crosslinking (corresponding to an average mesh size close to the diameter of a tracer particle), the subdiffusive regime at intermediate times becomes more pronounced before reaching the linear regime at longer times. Such a change in the dynamic features at high degree of crosslinking was interpreted in terms of trapping/caging within the polymer mesh at short times and infrequent jumps out of the cage at long times. The interpretation was confirmed by comparing the MSDs, van Hove correlation functions, and intermediate scattering functions, as well as by directly observing particle displacements during simulation trajectories.

Interestingly, the effect of increasing polymer volume fraction at constant number of crosslinks is somewhat counterintuitive. It is obvious that tracer diffusion becomes slower at higher polymer volume fractions due to stronger obstruction caused by a high concentration of polymer segments near the tracer particle. Nevertheless, the cage dynamics and hopping processes, as opposed to normal Brownian diffusion, were less pronounced at higher polymer volume fractions, despite the stronger obstruction effect. We conjectured that the restraints that were imposed by permanent crosslinks on the conformational relaxation of network strands are stronger at lower polymer volume fractions, due to the extended/stretched conformations of the network strands, than at higher polymer volume fractions with more relaxed conformations. Therefore, the trapping/caging of a tracer particle within the polymer mesh is more effective at lower polymer volume fractions, inhibiting normal Brownian diffusion, but allowing infrequent hopping processes out of the cage. This conjecture for stronger restraints at lower polymer volume fractions was confirmed by smaller fluctuations in mesh size at lower polymer volume fractions. It was concluded that the cage dynamics and hopping process are well correlated with small fluctuations in network meshes.

The models of homogeneous polymer networks that were considered in this work assumed the athermal condition at which the polymer segments interact via excluded-volume interactions. In addition, the change in polymer volume fraction was induced by changing the system volume while keeping the number of polymer segments constant. In real polymer gels, the interaction between polymer segments may play an important role in driving the volume phase transitions as well as determining the equilibrium structure and dynamics of the network meshes. Furthermore, the attractive interactions between a tracer particle and the polymer segments can be even more important to tracer diffusion [8,19]. Despite these limitations of the models, this work clearly reveals the importance of the mesh size fluctuations in determining the transport of a tracer particle embedded in tightly meshed polymer networks. A small fluctuation in mesh size induces trapping of the particle in the meshes and results in the hopping mechanism of a tracer particle that has led to the non-Gaussian displacement distributions and the two-step decay of the intermediate scattering functions.

## Supplementary Materials

The following are available online at s1, Figure S1: Simulation systems with varying degrees of crosslinking and swelling, Figure S2: Mean-square displacement, $\langle \Delta r^{2}\left(t\right)\rangle$, for various $\xi /\sigma_{tr}$ with respect to *t* for ϕ = 0.1 and 0.3, Figure S3: $4\pi r^{2}G_{s}\left(r,t\right)$ corresponding to $G_{s}\left(r,t\right)$ in Figure 4A,B, Figure S4: Representative trajectories of a tracer particle performing diffusive and hopping motions, respectively, at volume fraction ϕ = 0.2, Figure S5: Representative trajectories of a tracer particle in polymer networks with similar values of $\xi /\sigma_{tr}\approx 1.37$.

## Abbreviations

The following abbreviations are used in this manuscript:

BD	Brownian dynamics
MSD	Mean square displacments
LJ	Lennard Jones
FENE	Finite Extension nonlinear elastic
