# Supplementary material for: Tracer Diffusion in Tightly-Meshed Homogeneous Polymer Networks: A Brownian Dynamics Simulation Study
Source: Polymers (Basel). 2020 Sep 11;12(9):2067. doi: 10.3390/polym12092067 (PMC7569880; doi:10.3390/polym12092067)
Supplement: Supplementary file 1 [file polymers-12-02067-s001.pdf]

Supplementary Material

Tracer diffusion in tightly-meshed homogeneous polymer networks: a Brownian dynamics simulation study

Hyun Woo Cho[1,†], Haein Kim[2,†], Bong June Sung[1,*] , and Jun Soo Kim[2,*]

[1] *Department of Chemistry, Sogang University, Seoul 04107, Republic of Korea*
[2] *Department of Chemistry and Nanoscience, Ewha Womans University, Seoul 03760, Republic of Korea*

[*] Correspondence: jkim@ewha.ac.kr (J.S.K.), bjsung@sogang.ac.kr (B.J.S.)
[†] These authors contributed equally.

Contents

Figure S1. Simulation systems with varying degrees of crosslinking and swelling

Figure S2. Mean-square displacement, $\langle \Delta r^2(t) \rangle$, for various ξ/σ_{tr} with respect to t for $\phi =$ 0.1 and 0.3

Figure S3. $4\pi r^2 G_s(r,t)$ corresponding to $G_s(r,t)$ presented in Figure 4 (A) and (B)

Figure S4. Representative trajectories of a tracer particle performing diffusive and hopping motions, respectively, at volume fraction $\phi = 0.2$

Figure S5. Representative trajectories of a tracer particle in polymer networks with similar values of $\xi/\sigma_{tr} \approx 1.37$

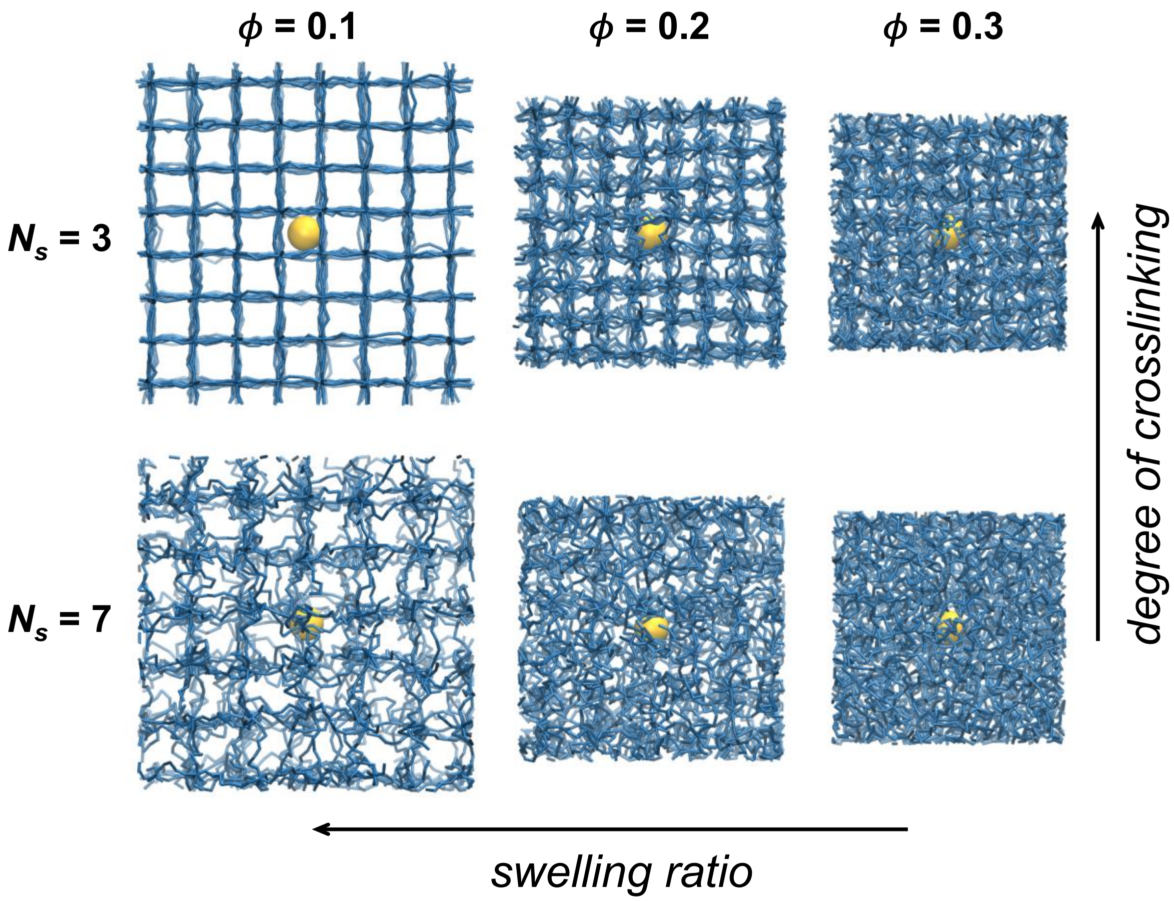

Figure S1. Simulation systems with varying degrees of crosslinking and swelling. Degree of crosslinking decreases as the number of polymer segments between crosslinkers (N_s) increases while swelling ratio decreases as the volume fraction of polymer network (ϕ) increases. The periodic boundary condition is applied in all directions and linear chains are self-connected to maintain the network structure. The size of a unit simulation cell is reduced for the same polymer network to increase the volume fraction from 0.1 to 0.2 and to 0.3. The cell size is around 30, 24, and 20 for the volume fractions of 0.1, 0.2, and 0.3, respectively.

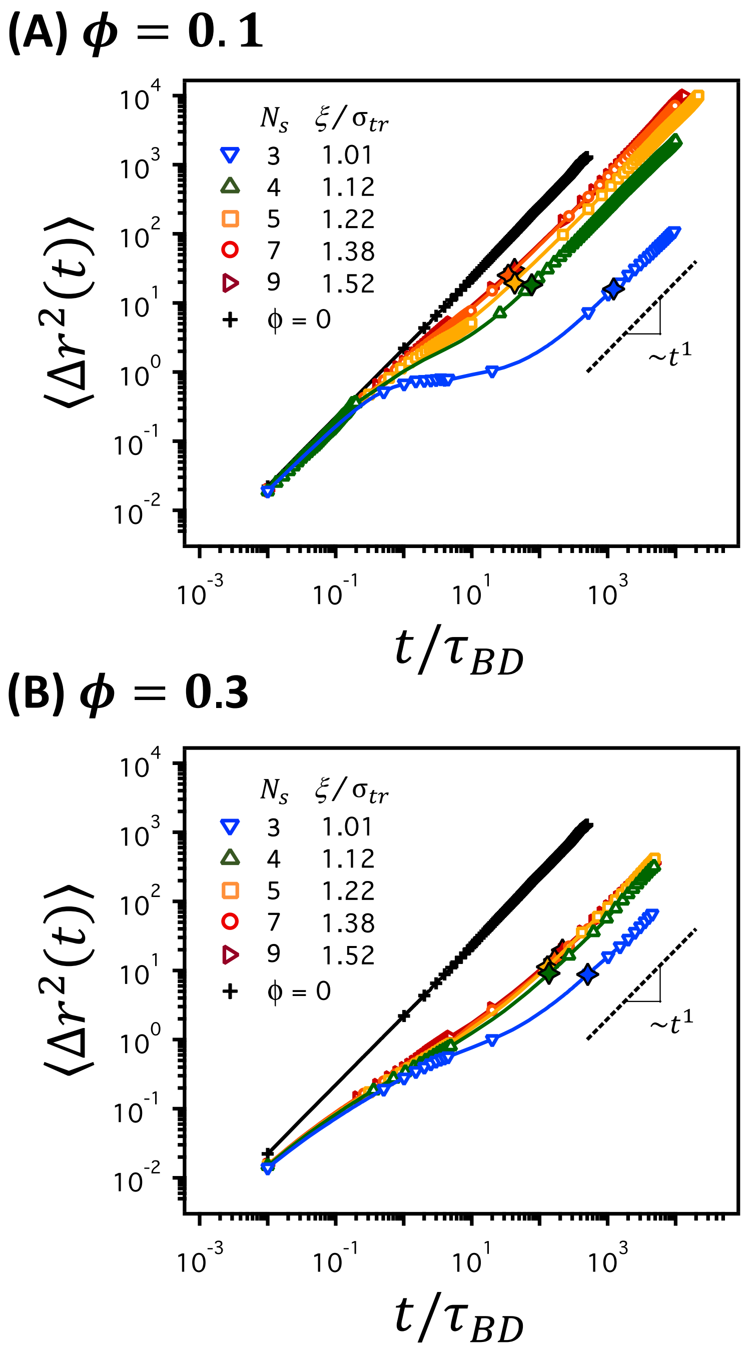

Figure S2. Mean-square displacement, $\langle \Delta r^2(t) \rangle$, for various ξ/σ_{tr} with respect to t for $\phi = 0.1$ and 0.3. The black open circles represent $\langle \Delta r^2(t) \rangle$ of the reference system for $\phi = 0$. The open stars mark the diffusion time scale τ_D. The definition of τ_D is described in the text. The line of $\sim t$ (the black dashed line) indicates that $\langle \Delta r^2(t) \rangle$ of all ξ/σ_{tr} reaches the linear regime for $t > \tau_D$.

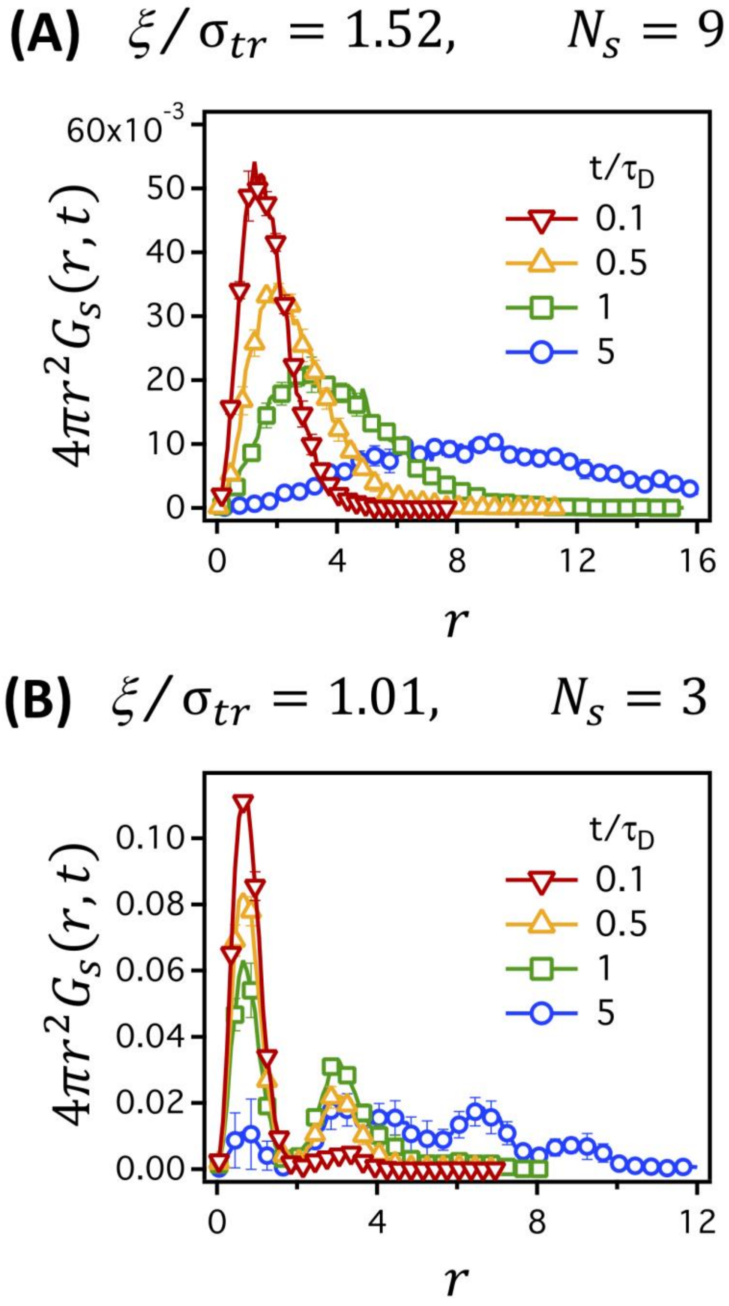

Figure S3. $4\pi r^2 G_s(r,t)$ **corresponding to** $G_s(r,t)$ **presented in Figure 4 (A) and (B).**

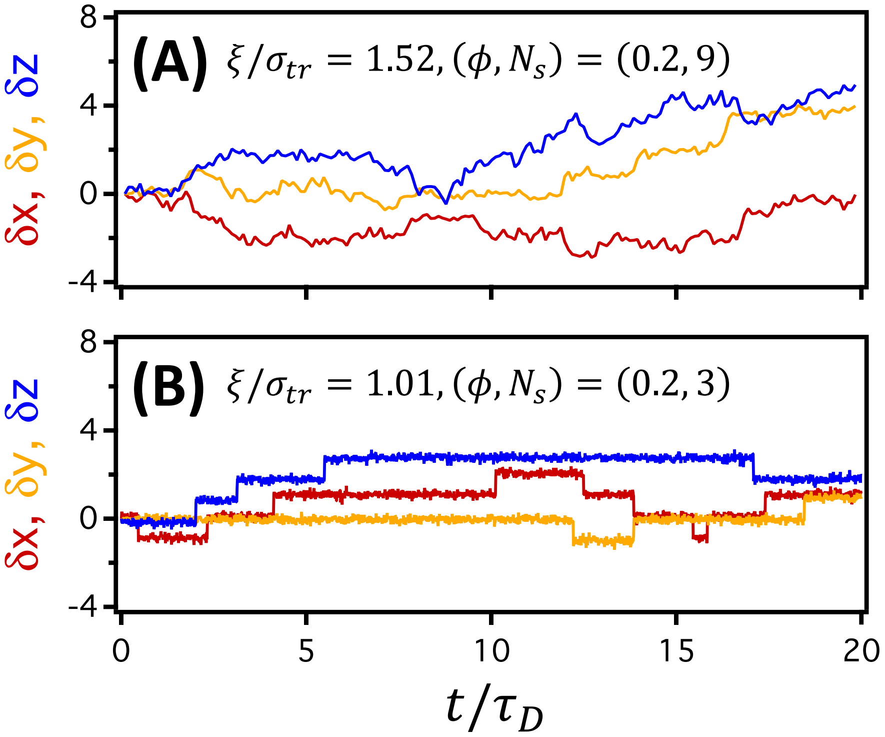

Figure S4. Representative trajectories of a tracer particle performing diffusive and hopping motions, respectively, at volume fraction $\boldsymbol{\phi = 0.2}$. (A) In the network with $N_s = 9$ or $\xi/\sigma_{tr} = 1.52$. (B) In the network with $N_s = 3$ or $\xi/\sigma_{tr} = 1.01$. δx, δy, and δz refer to the displacement of a tracer particle in x, y, and z directions, respectively, in unit of σ_{tr}. τ_D is the crossover time distinguishing subdiffusion and normal diffusion, as indicated in Fig. 3 of the main text.

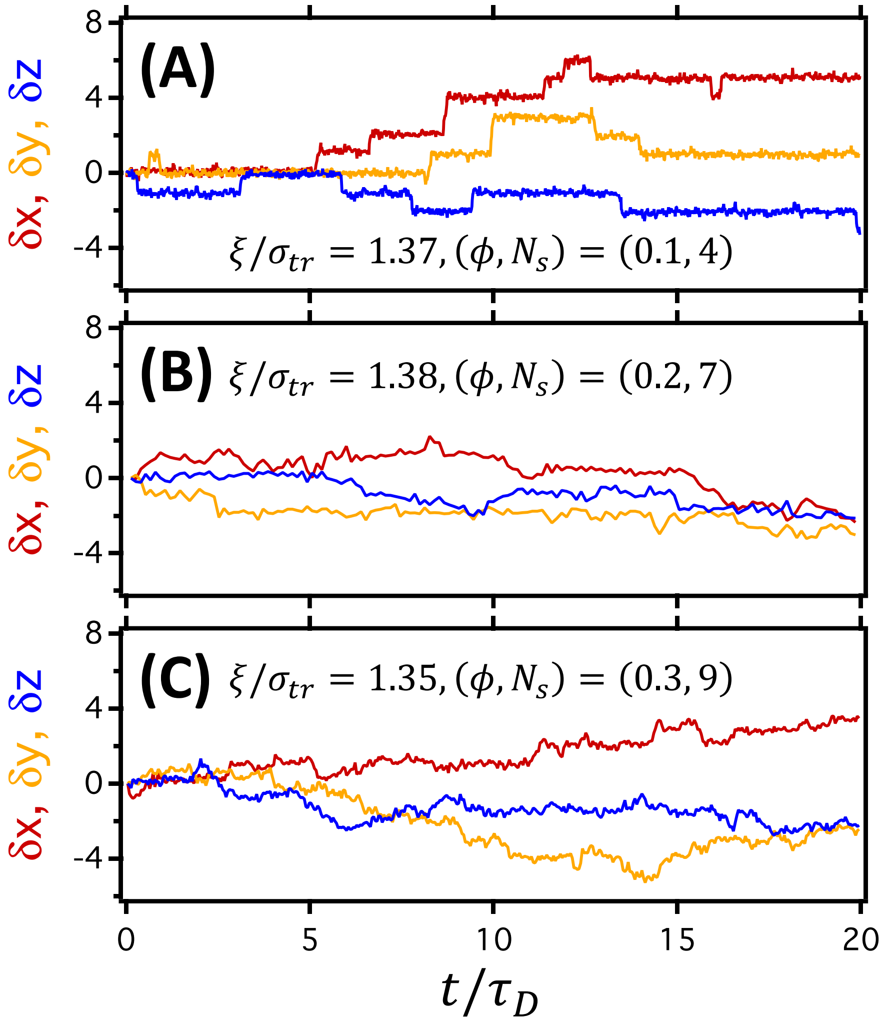

Figure S5. Representative trajectories of a tracer particle in polymer networks with similar values of $\xi/\sigma_{tr} \approx 1.37$. (A) In the network with $\phi = 0.1$ and $N_s = 4$. (B) In the network with $\phi = 0.2$ and $N_s = 7$. (C) In the network with $\phi = 0.3$ and $N_s = 9$. δx, δy, and δz refer to the displacement of a tracer particle in x, y, and z directions, respectively, in unit of σ_{tr}. τ_D is the time scale required for diffusive motion as indicated in Fig. 3 of the main text.
